# Supplementary material for: Taxonomic Identification and Molecular DNA Barcoding of Collected Wild-Growing Orchids Used Traditionally for Salep Production
Source: Plants (Basel). 2023 Aug 24;12(17):3038. doi: 10.3390/plants12173038 (PMC10489719; doi:10.3390/plants12173038)
Supplement: Supplementary file 1 [file plants-12-03038-s001.zip › plants-2483093-supplementary.pdf]

Supplementary Table S1. Collection information for different Greek wild-growing species of salep orchids (Orchidaceae) assigned with International Plant Exchange Network (IPEN) accession numbers and combined taxonomic and/ or molecular identification based on analysis of morphological data and BLAST tool results, respectively.

Collection information of Greek wild-growing species of Orchidaceae						Species composition based on taxonomic identification combined with molecular barcoding data		
**Serial No.**	**IPEN number**	**Collection area**	**Date**	**Latitude (North)**	**Longitude (East)**	**Taxonomic identification**	**Blast-based identification/ Molecular barcoding *ITS/matK/psbA-trnH***	**Consensus**
1	BBGK-1-18,6097 -1	Mountain areas of Kozani, West Macedonia, Greece	June 2018	40.300581	21.789813	*Dactylorhiza* sp.	*Dactylorhiza* sp.	*Dactylorhiza* sp.
2	BBGK-1-18,6097 -2	Mountain areas of Kozani, West Macedonia, Greece	June 2018	40.300581	21.789813	*Dactylorhiza sambucina*	*Dactylorhiza* sp. (*D. sambucina*/ *D. viridis*)	*Dactylorhiza sambucina*
3	BBGK-1-18,6097 -3	Mountain areas of Kozani, West Macedonia, Greece	June 2018	40.300581	21.789813	*Anacamptis* sp.	*Anacamptis* sp.	*Anacamptis* sp.
4	BBGK-1-18,6097 -4	Mountain areas of Kozani, West Macedonia, Greece	June 2018	40.300581	21.789813	*Anacamptis morio* subsp. *caucasica*	*Anacamptis* sp. (*A. morio*)	*Anacamptis morio* subsp. *caucasica*
5	BBGK-1-18,6097 -5	Mountain areas of Kozani, West Macedonia, Greece	June 2018	40.300581	21.789813	*Anacamptis morio* subsp. *caucasica*	*Anacamptis* sp.	*Anacamptis morio* subsp. *caucasica*
6	BBGK-1-18,6097 -6	Mountain areas of Kozani, West Macedonia, Greece	June 2018	40.300581	21.789813	*Anacamtis morio* subsp. *caucasica*	*Anacamptis morio*	*Anacamptis morio* subsp. *caucasica*
7	BBGK-1-18,6097-7	Mountain areas of Kozani, West Macedonia, Greece	June 2018	40.300581	21.789813	*Dactylorhiza sambucina*	*Dactylorhiza* sp.	*Dactylorhiza sambucina*

8	BBGK-1-18,6097 -8	Mountain areas of Kozani, West Macedonia, Greece	June 2018	40.300581	21.789813	*Anacamptis morio* subsp. *caucasica*	*Anacamptis* sp. *(A. morio)*	*Anacamptis morio* subsp. *caucasica*
9	BBGK-1-18,6097 -9	Mountain areas of Kozani, West Macedonia, Greece	June 2018	40.300581	21.789813	*Dactylorhiza* sp.	*Dactylorhiza* sp.	*Dactylorhiza* sp.
10	BBGK-1-18,6097 -10	Mountain areas of Kozani, West Macedonia, Greece	June 2018	40.300581	21.789813	*Dactylorhiza sambucina*	*Dactylorhiza sambucina*	*Dactylorhiza sambucina*
11	BBGK-1-18,6097 -11	Mountain areas of Kozani, West Macedonia, Greece	June 2018	40.300581	21.789813	*Anacamptis morio* subsp. *caucasica*	*Anacamptis morio*	*Anacamptis morio* subsp. *caucasica*
12	BBGK-1-18,6097 -12	Mountain areas of Kozani, West Macedonia, Greece	June 2018	40.300581	21.789813	*Anacamptis morio* subsp. *caucasica*	*Anacamptis* sp. *(A. morio)*	*Anacamptis morio* subsp. *caucasica*
13	BBGK-1-18,6097 -13	Mountain areas of Kozani, West Macedonia, Greece	June 2018	40.300581	21.789813	*Anacamptis morio* subsp. *caucasica*	*Anacamptis* sp. *(A. morio)*	*Anacamptis morio* subsp. *caucasica*
14	BBGK-1-18,6097 -14	Mountain areas of Kozani, West Macedonia, Greece	June 2018	40.300581	21.789813	*Anacamptis morio* subsp. *caucasica*	*Anacamptis morio*	*Anacamptis morio* subsp. *caucasica*
15	BBGK-1-18,6097 -15	Mountain areas of Kozani, West Macedonia, Greece	June 2018	40.300581	21.789813	*Anacamptis morio* subsp. *caucasica*	*Anacamptis* sp.	*Anacamptis* subsp. *caucasica*
16	BBGK-1-18,6067 -16	Mountain areas of Kozani, West Macedonia, Greece	June 2018	40.300581	21.789813	*Anacamptis morio* subsp. *caucasica*	*Anacamptis* sp. *(A. morio)*	*Anacamptis morio* subsp. *caucasica*
17	BBGK-1-18,6097 -17	Mountain areas of Kozani, West Macedonia, Greece	June 2018	40.300581	21.789813	*Anacamptis morio* subsp. *caucasica*	*Anacamptis morio*	*Anacamptis morio* subsp. *caucasica*

18	BBGK-1-18,6097 -18	Mountain areas of Kozani, West Macedonia, Greece	June 2018	40.300581	21.789813	*Dactylorhiza* sp. *(D. sambucina)*	*Dactylorhiza* sp. *(D. sambucina/ D. viridis)*	*Dactylorhiza. sambucina*
19	BBGK-1-18,6097 -19	Mountain areas of Kozani, West Macedonia, Greece	June 2018	40.300581	21.789813	*Anacamptis morio* subsp. *caucasica*	*Anacamptis morio*	*Anacamptis morio* subsp. *caucasica*
20	BBGK-1-18,6097 -20	Mountain areas of Kozani, West Macedonia, Greece	June 2018	40.300581	21.789813	*Dactylorhiza* sp.	*Dactylorhiza* sp.	*Dactylorhiza* sp.
21	BBGK-1-18,6097 -21	Mountain areas of Kozani, West Macedonia, Greece	June 2018	40.300581	21.789813	*Anacamptis morio* subsp. *caucasica*	*Anacamptis morio*	*Anacamptis morio* subsp. *caucasica*
22	BBGK-1-18,6097 -22	Mountain areas of Kozani, West Macedonia, Greece	June 2018	40.300581	21.789813	*Anacamptis morio* subsp. *caucasica*	*Anacamptis* sp. *(A. Morio)*	*Anacamptis morio* subsp. *caucasica*
23	BBGK-1-18,6097 -23A	Mountain areas of Kozani, West Macedonia, Greece	June 2018	40.300581	21.789813	*Dactylorhiza sambucina*	*Dactylorhiza sambucina*	*Dactylorhiza sambucina*
24	BBGK-1-18,6097 -23B	Mountain areas of Kozani, West Macedonia, Greece	June 2018	40.300581	21.789813	*Dactylorhiza sambucina*	*Dactylorhiza sambucina*	*Dactylorhiza sambucina*
25	BBGK-1-18,6097 -24	Mountain areas of Kozani, West Macedonia, Greece	June 2018	40.300581	21.789813	*Dactylorhiza sambucina*	*Dactylorhiza sambucina/ D. incarnata*	*Dactylorhiza* sp. (*D. sambucina)*
26	BBGK-1-18,6097 -26	Mountain areas of Kozani, West Macedonia, Greece	June 2018	40.300581	21.789813	-	*Dactylorhiza* sp. *(D. maculata/ D. sambucina)*	*Dactylorhiza maculata / Dactylorhiza sambucina*
27	BBGK-1-18,6097 -27	Mountain areas of Kozani, West Macedonia, Greece	June 2018	40.300581	21.789813	-	*Dactylorhiza maculata*	*Dactylorhiza maculata*
28	BBGK-1-18,6097 -28	Mountain areas of Kozani, West	June 2018	40.300581	21.789813	*Dactylorhiza sambucina*	-	*Dactylorhiza sambucina*

		Macedonia, Greece						
29	BBGK-1-18,6097 -29	Mountain areas of Kozani, West Macedonia, Greece	June 2018	40.300581	21.789813	-	*Dactylorhiza* sp. (*D. maculata*/ *D. viridis*)	*Dactylorhiza maculata*
30	BBGK-1-18,6097 -30	Mountain areas of Kozani, West Macedonia, Greece	June 2018	40.300581	21.789813	*Dactylorhiza* sp.	*Dactylorhiza* sp.	*Dactylorhiza* sp.
31	BBGK-1-18,6097 -32	Mountain areas of Kozani, West Macedonia, Greece	June 2018	40.300581	21.789813	*Dactylorhiza* sp.	*Dactylorhiza* sp.	*Dactylorhiza* sp.
32	BBGK-1-18,6097-34	Mountain areas of Kozani, West Macedonia, Greece	June 2018	40.300581	21.789813	*Dactylorhiza* sp.	*Dactylorhiza* sp. (*D. viridis*)	*Dactylorhiza* sp.
33	BBGK-1-18,6097-35	Mountain areas of Kozani, West Macedonia, Greece	June 2018	40.300581	21.789813	-	*Dactylorhiza* sp. (*D. viridis*)	*Dactylorhiza* sp.
34	BBGK-1-18,6097-38	Mountain areas of Kozani, West Macedonia, Greece	June 2018	40.300581	21.789813	-	*Dactylorhiza* sp. (*D. viridis*)	*Dactylorhiza* sp.
35	BBGK-1-19,402	Republic of North Macedonia	September 2019	41.99646	21.43141	*Anacamptis pyramidalis*	*Anacamptis pyramidalis*	*Anacamptis pyramidalis*
36	BBGK-1-20,518	Kastoria, Greece	July 2020	40.520744	21.271206	-	*Dactylorhiza* sp. (*D. viridis*)	*Dactylorhiza* sp.
37	BBGK-1-20,519	Kastoria, Greece	July 2020	40.520744	21.271206	-	*Anacamptis* sp. (*A. morio*)	*Anacamptis sp.*
38	BBGK-1-21,113	Grevena Vasilitsa, Mt Lygkos, Annitsa monument, Dotsiko to Samarina, Greece	June 2021	39.9	21.1167	*Dactylorhiza sambucina*	*Dactylorhiza* sp. (*D. sambucina*)	*Dactylorhiza* sp. (*D. sambucina*)

39	BBGK-1-21,119	Fokida Tristeno, Greece	June 2021	38.606773	22.054661	*Dactylorhiza maculata* subsp. *saccifera*	*Dactylorhiza* sp. *(D. maculata* subsp. *fuchsii/D. maculata)*	*Dactylorhiza maculata* subsp. *saccifera*
40	BBGK-1-21,146	Mt Ambelos, Samos, South Aegean, Greece	June 2021	37.7994073	26.799683	*Dactylorhiza majalis* subsp. *pythagorae*	*Dactylorhiza* sp.	*Dactylorhiza majalis* subsp. *pythagorae*
41	BBGK-1-21,236	Smolikas, Ioannina, Greece	July 2021	40.0916663	20.921663	*Dactylorhiza* sp.	*Dactylorhiza* sp. (*D. maculata* subsp. *fuchsii/ D. maculata*)	*Dactylorhiza maculata* subsp. *saccifera*
42	BBGK-1-21,237	Smolikas, Ioannina, Greece	July 2021	40.0916663	20.921663	*Dactylorhiza* sp. (*D. maculata* subsp. *saccifera*		*Dactylorhiza maculata*
43	BBGK-1-21,238	Smolikas, Ioannina, Greece	July 2021	40.0916663	20.921663	*Dactylorhiza maculata* subsp. *saccifera*	*Dactylorhiza* sp. *(D. maculata)*	*Dactylorhiza maculata* subsp. *saccifera*
44	BBGK-1-21,239	Smolikas, Ioannina, Greece	July 2021	40.0916663	20.921663	*Orchis* sp.	*Orchis* sp. (*O. pallens*)	*Orchis pallens*
45	BBGK-1-21,240	Smolikas, Ioannina, Greece	July 2021	40.0916663	20.921663	*Orchis* sp.	*Orchis* sp. (*O. pallens*)	*Orchis pallens*
46	BBGK-1-21,241	Smolikas, Ioannina, Greece	July 2021	40.0916663	20.921663	*Orchis* sp.	*Orchis* sp. (*O. pallens*)	*Orchis pallens.*
47	BBGK-1-21,242	Smolikas, Ioannina, Greece	July 2021	40.0916663	20.921663	*Orchis* sp.	*Orchis* sp. (*O. pallens*)	*Orchis pallens*
48	BBGK-1-21,243	Smolikas, Ioannina, Greece	July 2021	40.0916663	20.921663	*Orchis* sp.	*Orchis* sp. *(O. quadripuncata)*	*Orchis quadripuncata*
49	BBGK-1-21,244	Smolikas, Ioannina, Greece	July 2021	40.0916663	20.921663	*Orchis* sp.	*Orchis* sp. (*O. pallens*)	*Orchis pallens*
50	BBGK-1-22,59	Grevena, Valia Kalda, West Macedonia, Greece	June 2022	39.898260	21.141416	*Dactylorhiza sambucina*	*Dactylorhiza* sp.	*Dactylorhiza sambucina*
51	BBGK-1-22,60	Grevena, Valia Kalda, West Macedonia, Greece	June 2022	39.898260	21.141416	*Dactylorhiza maculata* subsp. *saccifera*	*Dactylorhiza maculata*	*Dactylorhiza maculata* subsp. *saccifera*
52	BBGK-1-22,61	Grevena, Valia Kalda, West Macedonia, Greece	June 2022	39.898260	21.141416	*Himantoglossum calcaratum* subsp. *rumelicum*	*Himantoglossum* sp.	*Himantoglossum calcaratum* subsp. *rumelicum*

53	BBGK-1-22,62	Grevena, Valia Kalda, West Macedonia, Greece	June 2022	39.898260	21.141416	*Dactylorhiza maculata* subsp. *saccifera*	*Dactylorhiza maculata*	*Dactylorhiza maculata* subsp. *saccifera*

Supplementary Table S2. Percentage identities of BLAST results for the 51 samples used for molecular barcoding indicated with their IPEN (International Plant Exchange Network) accession numbers. Low BLAST similarities appear in red. Asterisks (*) denote divergent results (different genera). Bold letters in samples 48 to 51 are *rbcL* barcoding marker results.

No.	IPEN Accession number	*ITS*	*matK*	*trnH-psbA* or *rbcL*
1	BBGK-1-18,6097 -1	98.9	99.7	x
2	BBGK-1-18,6097 -2	98.9	99.3	97.2
3	BBGK-1-18,6097 -3	x	98.5	94.7
4	BBGK-1-18,6097 -4	x	97.9	93.7* (*Ophrys* sp.)
5	BBGK-1-18,6097 -5	x	99.0	98.2
6	BBGK-1-18,6097 -6	99.0	98.6	x
7	BBGK-1-18,6097-7	99.6	x	97.0
8	BBGK-1-18,6097 -8	x	98.1	96.5
9	BBGK-1-18,6097 -9	x	97.7	x
10	BBGK-1-18,6097 -10	99.9	99.5	98.1
11	BBGK-1-18,6097 -11	97.6	98.6	98.1
12	BBGK-1-18,6097 -12	99.2	98.4	x
13	BBGK-1-18,6097 -13	99.0	98.0	95.3* (*Ophrys* sp.)
14	BBGK-1-18,6097 -14	98.8	97.8	98.4
15	BBGK-1-18,6097 -15	x	x	98.1
16	BBGK-1-18,6067 -16	98.6	97.9	x
17	BBGK-1-18,6097 -17	96.3	98.2	94.8* (*Ophrys* sp.)
18	BBGK-1-18,6097 -18	99.9	99.0	98.2
19	BBGK-1-18,6097 -19	99.3	98.6	97.4
20	BBGK-1-18,6097 -20	x	99.7	98.3
21	BBGK-1-18,6097 -21	98.8	x	95.6
22	BBGK-1-18,6097 -22	95.2	97.4	97.7
23	BBGK-1-18,6097 -23A	99.7	98.3	96.0
24	BBGK-1-18,6097 -23B	100.0	99.0	98.0
25	BBGK-1-18,6097 -24	100.0	99.7	97.2
26	BBGK-1-18,6097 -26	98.9	99.9	x
27	BBGK-1-18,6097 -27	99.5	99.7	98.1
28	BBGK-1-18,6097 -29	99.1	99.0	x
29	BBGK-1-18,6097 -30	97.8	99.1	x

30	BBGK-1-18,6097 -32	98.9	99.7	x
31	BBGK-1-18,6097-34	99.4	98.1	x
32	BBGK-1-18,6097-35	99.0	99.2	x
33	BBGK-1-18,6097-38	96.0	99.1	x
34	BBGK-1-19,402	98.9	98.9	93.1* (*Heminium* sp.)
35	BBGK-1-20,518	98.5	98.6	x
36	BBGK-1-20,519	x	97.5	x
37	BBGK-1-21,113	99.4	98.1	97.9
38	BBGK-1-21,119	97.5	99.9	
39	BBGK-1-21,146	98.2	x	x
40	BBGK-1-21,236	99.6	98.1	96.0
41	BBGK-1-21,238	98.7	99.0	93.6
42	BBGK-1-21,239	98.8	97.2	94.5* (*Heminium* sp.)
43	BBGK-1-21,240	99.2	98.3	93.2
44	BBGK-1-21,241	97.8	96.4	x
45	BBGK-1-21,242	98.3	97.9	90.6* (*Heminium* sp.)
46	BBGK-1-21,243	98.4	98.6	91.8* (*Heminium* sp.)
47	BBGK-1-21,244	98.8	98.2	91.3* (*Heminium* sp.)
48	BBGK-1-22,59	x	96.7	**x**
49	BBGK-1-22,60	98.3	99.4	**98.7**
50	BBGK-1-22,61	97.8	97.8	**99.0**
51	BBGK-1-22,62	98.5	99.1	**x**

Supplementary Table S3. NCBI GenBank-deposited accession numbers for 11 Orchidaceae species.

Species	NCBI GenBank Accession Number
Gymnadenia orchidis	MF945491.1
Gymnadenia sp.	MF945501.1
Platanthera chlorantha	EF612531.1
Dactylorhiza baltica	MF945403.1
Dactylorhiza viridis	KJ452797.1
Platanthera sparsiflora	MF945504.1
Platanthera leptocaulon	KJ452824.1
Diphylax contigua	KJ452845.1

Diphylax uniformis	JN696434.1
Orchis quadripunctata	AY368385.1
Orchis mascula	JN896032.1

Supplementary Table S4. Sequences of the PCR primers used in the experiments.

Primers Used	Forward Primer	Reverse Primer
ITS (Tamura et al., 2021) [45]	ATGCGATACTTGGTGTGAAT	GACGCTTCTCCAGACTACAAT
matK (Cuénoud et al., 2002) [47]	CGATCTATTCATTCAATATTTC	TCTAGCACACGAAAGTCGAAGT
trnH-psbA (Costion et al., 2011) [46]	GTTATGCATGAACGTAATGCTC	CGCGCATGGTGGATTCACAATCC
Rbcl (Ismail et al., 2020) [48]	ATGTCACCACAAACAGAAAC	TCGCATGTACCTGCAGTAGC

References [45, 46, 47, 48] presented herein in the "Supplementary Materials" (Table S4) are cited in the main text

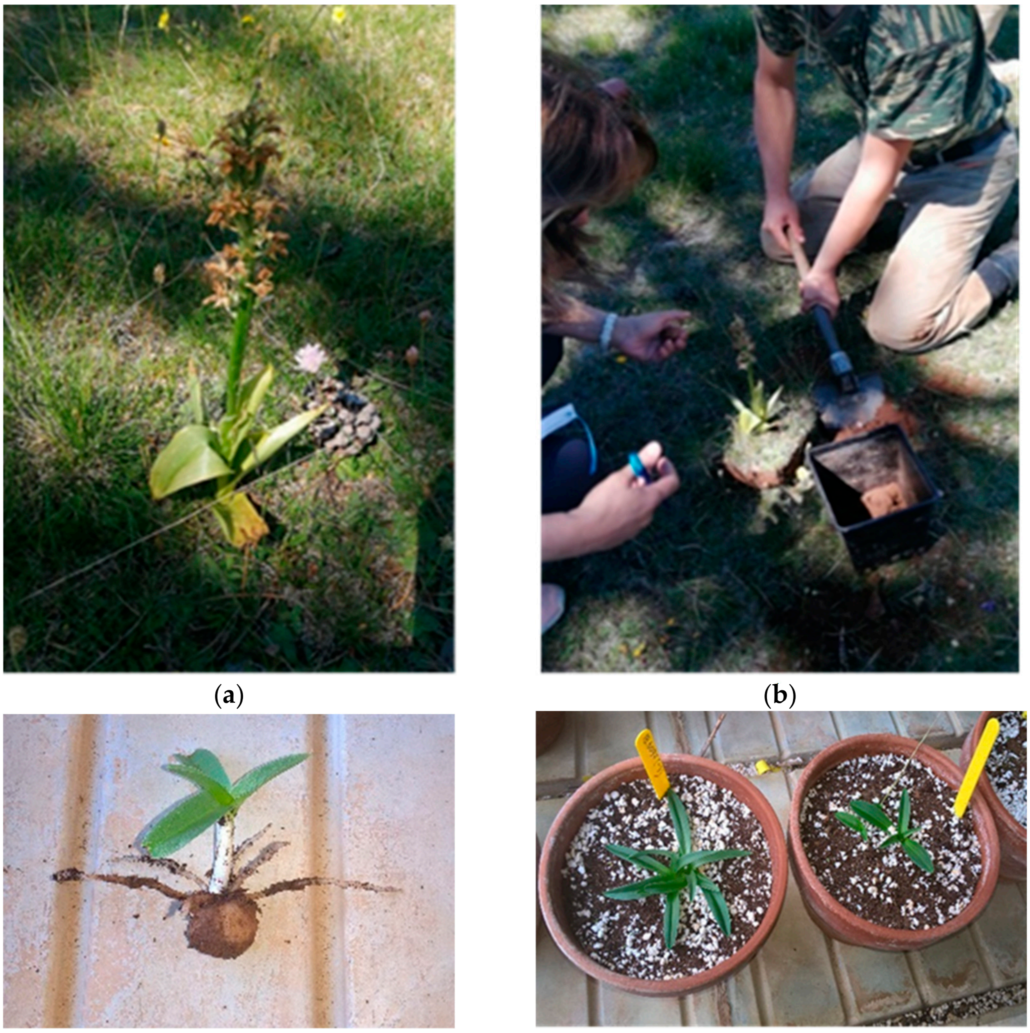

(**a**) (**b**)

(**c**) (**d**)

Supplementary Figure S1. Collection and handling of wild-growing Greek orchid samples during ex situ conservation at the premises of the Institute of Plant Breeding and Genetic Resources, Agricultural Organization—Demeter (Thermi, metropolitan Thessaloniki, Greece): (**a** and **b**) Collection of wild-growing *Orchis* sp. individuals from Mt. Smolikas, northwestern Greece for ex situ conservation; (**c**) Separation of individual orchid tubers; (**d**) Potted individual orchid tubers.

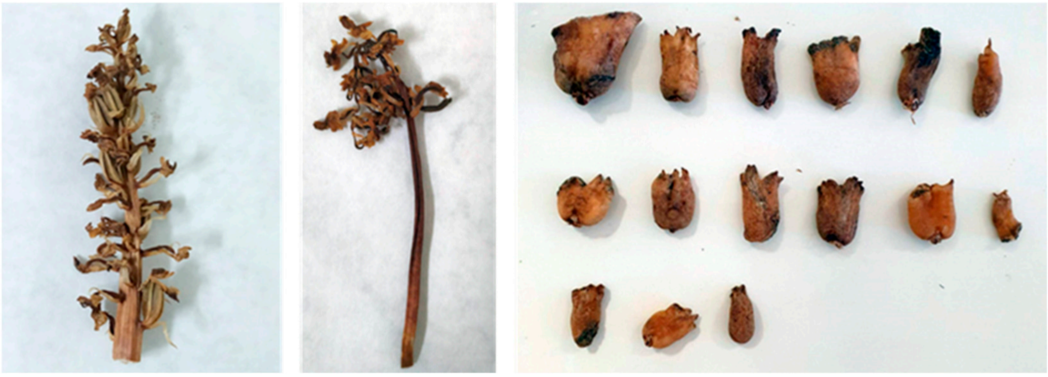

(**a**) (**b**) (c)

Supplementary Figure S2. (**a**) Dried inflorescences; (**b**) above-ground parts; and (**c**) tubers used for DNA extraction.
